# In Standing, Corticospinal Excitability Is Proportional to COP Velocity Whereas M1 Excitability Is Participant-Specific

**DOI:** 10.3389/fnhum.2018.00303

**Published:** 2018-07-30

**Authors:** Tulika Nandi, Claudine J. C. Lamoth, Helco G. van Keeken, Lisanne B. M. Bakker, Iris Kok, George J. Salem, Beth E. Fisher, Tibor Hortobágyi

**Affiliations:** ^1^Center for Human Movement Sciences, University of Groningen, University Medical Center Groningen, Groningen, Netherlands; ^2^Division of Biokinesiology and Physical Therapy, University of Southern California, Los Angeles, CA, United States

**Keywords:** standing, sway, M1 excitability, corticospinal excitability, task difficulty

## Abstract

Reductions in the base of support (BOS) make standing difficult and require adjustments in the neural control of sway. In healthy young adults, we determined the effects of reductions in mediolateral (ML) BOS on peroneus longus (PL) motor evoked potential (MEP), intracortical facilitation (ICF), short interval intracortical inhibition (SICI) and long interval intracortical inhibition (LICI) using transcranial magnetic stimulation (TMS). We also examined whether participant-specific neural excitability influences the responses to increasing standing difficulty. Repeated measures ANOVA revealed that with increasing standing difficulty MEP size increased, SICI decreased (both *p* < 0.05) and ICF trended to decrease (*p* = 0.07). LICI decreased only in a sub-set of participants, demonstrating atypical facilitation. Spearman’s Rank Correlation showed a relationship of ρ = 0.50 (*p* = 0.001) between MEP size and ML center of pressure (COP) velocity. Measures of M1 excitability did not correlate with COP velocity. LICI and ICF measured in the control task correlated with changes in LICI and ICF, i.e., the magnitude of response to increasing standing difficulty. Therefore, corticospinal excitability as measured by MEP size contributes to ML sway control while cortical facilitation and inhibition are likely involved in other aspects of sway control while standing. Additionally, neural excitability in standing is determined by an interaction between task difficulty and participant-specific neural excitability.

## Introduction

Mechanical challenges and sensory manipulations of standing balance increase the spontaneous movements of the center of mass (Prieto et al., 1996; Amiridis et al., 2003). Reductions in the base of support (BOS) make it difficult to maintain balance and require adjustments in the neural control of center of pressure (COP). In response to manipulations that challenge standing balance, fronto-parietal alpha and theta EEG power increases, indicating an increase in cortical activity (Slobounov et al., 2009; Goh et al., 2017). Also, corticospinal excitability and primary motor cortex (M1) inhibition measured by transcranial magnetic stimulation (TMS), increases and decreases respectively (Tokuno et al., 2009; Baudry et al., 2014; Papegaaij et al., 2014, 2016; Nandi et al., 2018). Presumably such neural adjustments tune muscle contractions, adjust COP dynamics and consequently center of mass sway, thereby ensuring that balance is maintained.

In contrast to anteroposterior (AP) and direction non-specific manipulations, we recently demonstrated that mediolateral (ML) manipulations of BOS produce correlated changes in the neural excitability of the tibialis anterior (TA) and COP velocity in young adults (Nandi et al., 2018). These findings are in line with EEG observations indicating that active neural control is greater during ML compared to AP sway (Slobounov et al., 2008). However, the correlations were weak, possibly because the TA is also a primary dorsiflexor, which is essential for AP control. The peroneus longus (PL) may be physiologically and anatomically a more accurate target than the TA to determine the effects of ML manipulations on neural control of standing sway. Both PL and TA activity increase with ML sway, but PL activity is necessary only for ML control since plantarflexor forces are generated primarily by soleus and gastrocnemius (Sozzi et al., 2013). Additionally, impaired PL control has been implicated in postural deficits associated with ankle instability (Konradsen and Ravn, 1990; Kim et al., 2012). However, neural excitability of the PL has not been examined in standing (Luc et al., 2014). Thus, we determined the effects of ML standing task difficulty manipulation on corticospinal and M1 excitability of the PL, in healthy young adults. We expected to find an increase in corticospinal excitability and decrease in M1 GABAa inhibition and M1 facilitation, correlated with the increase in ML COP velocity as task difficulty increases. This expectation would lend support to the idea that active neural control, particularly cortical involvement in ML sway control, increases with task difficulty. Also, we examined, for the first time, M1 GABAb inhibition, which shows distinct task-specific modulation compared to GABAa inhibition (Opie et al., 2015).

Numerous studies have reported large between-participant variation in neural excitability of hand and leg muscles using TMS (Hamada et al., 2012; Heise et al., 2013; Fedele et al., 2016; Nandi et al., 2018). Such variation is found despite high test-retest reliability (Orth et al., 2003) leading to the idea of participant-specific “intrinsic neural excitability” (Greenhouse et al., 2017). Therefore, we considered the so far overlooked possibility that the neural modulation in response to changing task difficulty is dependent on excitability measured in the control task. Specifically, we examined whether neural excitability in the control task, i.e., wide stance, predicts changes in excitability as standing difficulty increases. We manipulated task difficulty by decreasing the ML BOS (wide, narrow, tandem, one leg). To minimize variation due to unreliability and strengthen our inferences, we examined task-specific reliability of each outcome variable to guide the main analyses.

## Materials and Methods

### Participants

Fourteen healthy young adults aged 22.3 ± 1.7 years (mean ± SD, 12F) volunteered for the main study and data were acquired during a single 1.5 h long lab visit. Reliability of TMS outcomes was examined in another group of 15 young adults (22.1 ± 2.0, 11F) who visited the lab twice ~7 days apart. This study was carried out in accordance with the recommendations of the Medical Ethical Committee of the University Medical Center Groningen. The protocol was approved by the Medical Ethical Committee. All subjects gave written informed consent in accordance with the Declaration of Helsinki (World Medical Association, 2013). A safety questionnaire (Rossi et al., 2009) was used to exclude individuals with history of neurological or orthopedic disorders, seizures, head trauma; suspicion of pregnancy; metal implants or pacemakers; used medication known to lower seizure threshold or had blood relatives with a history of seizures. We also determined foot dominance (Hebbal and Mysorekar, 2006). Level of physical activity (Craig et al., 2003) and mobility (Guralnik et al., 1994) were measured to ensure that our study sample had relatively similar physical activity levels, which can affect balance, and consequently our outcome measures. No participants were excluded based on these data.

### Procedures

Measurement of TMS and COP outcomes was conducted in four tasks: (1) wide stance (feet shoulder width apart); (2) narrow stance (feet together); (3) tandem stance (dominant foot posterior), and (4) one leg stance (dominant foot). Participants wore socks and stood with arms crossed across the chest. Task order was randomized across participants, with 2–3 min of rest between tasks. Maximal voluntary contraction (MVC) was used to normalize and compare background EMG (bEMG) across tasks and participants. Two methods were used—manual resistance against ankle plantarflexion and eversion in sitting or heel rise while standing on one leg. Each method was repeated three times and the highest EMG obtained from all six trials was used as an estimate of MVC.

### Data Acquisition

Wireless sensors (dimensions—37*26*15 mm, electrode material—silver; Trigno™ Wireless System, Delsys, Natick, MA, USA) were used to record EMG from the dominant side PL. The signal was amplified 1000 times and sampled at 5000 Hz using data acquisition interface and software (Power 1401 and Signal v5.11, Cambridge Electronic Design Ltd, Cambridge UK).

Magnetic pulses were applied using two single-pulse magnetic stimulators (Magstim Model 2002, The Magstim Co., Whitland, UK), a Bistim module and a double cone coil (110 mm). Participants wore a cloth cap marked with a grid and the coil was moved in 1 cm increments to determine the hot-spot which was defined as the location where the largest and most consistent motor evoked potentials (MEPs) were obtained. The hot-spot was marked on the cap to ensure consistent positioning of the coil, which was held by the researcher. In standing, the active motor threshold (MT) was determined by systematically varying the stimulation intensity to find the lowest level of stimulator output at which 3 out of 5 MEPs had a peak-to-peak amplitude of at least 50 μV. For eliciting short interval intracortical inhibition (SICI, GABAa mediated) and intracortical facilitation (ICF), the conditioning and test pulse were set at 70% and 110% MT, respectively. For long interval intracortical inhibition (LICI, GABAb mediated), the conditioning and test pulse were set at 120% and 110% MT respectively. An inter-stimulus interval (ISI) of 3, 13 and 100 ms was used for SICI, ICF and LICI, respectively (Rossini et al., 2015). These parameters were chosen based on extensive pilot testing which examined SICI, LICI and ICF using different combinations of intensities and ISIs. Ten paired pulses each for the SICI, LICI and ICF protocols, and 10 single pulses at 110% MT were applied in random order. There was an 8–10 s interval between pulses (or pulse pairs).

COP location was calculated using force and moment data obtained using two force plates (Bertec 4060-08, Columbus, OH, USA) embedded in the floor, sampled at 200 Hz and acquired using a custom LabVIEW script (v2015, National Instruments, Austin, TX, USA).

### Data Analyses

Data were analyzed using Matlab (The Mathworks, Natick, MA, USA). EMG was bandpass filtered using a 4th order dual pass Butterworth filter with 10 Hz and 1000 Hz high and low pass cut-offs, respectively. For the MVC trials, data were smoothed with a moving average with 100 ms non-overlapping windows, the peak voltage was measured and the highest of six trials used as an estimate of peak muscle activation during MVC. bEMG was estimated as the mean rectified signal over a 100 ms window before the TMS pulse and expressed as %MVC. Additionally, bEMG area was calculated by integrating the rectified EMG in the same window. MEP peak-to-peak amplitude was estimated from unrectified EMG, in a 100 ms window after application of the TMS pulse. For determination of MEP area, the filtered EMG was rectified, MEP onset was detected, and the data were integrated over a 100 ms window starting at onset. Onset was automatically detected in a 100 ms window after the TMS pulse, if the signal exceeded a bEMG+2SD threshold for at least three data points while the preceding data point remained below the threshold. To improve accuracy, the detected onset was visually inspected and manually corrected when required. Normalized MEP area was calculated by subtracting the trial specific bEMG area from MEP area (Runnalls et al., 2014). For each of the three measures 8–10 trials each were averaged to obtain estimates of test MEP and conditioned MEPs for SICI, LICI and ICF. A few trials were discarded due to improper coil placement or technical errors in syncing the TMS data with force plate data. SICI, LICI and ICF were quantified as: Conditioned MEP/Test MEP * 100. Higher values indicate lower inhibition (SICI and LICI) and greater facilitation (ICF).

The COP location time series was low pass filtered using a 4th order Butterworth filter with a 5 Hz cut-off. Pilot testing showed that placement of the coil on the head alters COP velocity, even in the absence of stimulation. Therefore, COP data was extracted from a 2 s window before application of each pulse, when the coil was already positioned on the head. The distance between each pair of consecutive data points was summed to obtain the total distance and divided by time to obtain ML COP velocity. Velocity was averaged across 40 trials to obtain a single estimate for each standing task.

### Statistical Analyses

All statistical analyses were conducted using SPSS (Version 24, IBM Corp., Armonk, NY, USA). The Shapiro-Wilk test revealed that several variables were not normally distributed, and these were log transformed for further analyses. Reliability of the TMS outcomes was estimated using intra-class correlation coefficients (ICC). ICC(2,k) i.e., a two-way random effects model for averaged measures (averaged over 8–10 trials) was used (Weir, 2005). Categories of ICCs were based on a recent multi-center TMS reliability study: ICC >0.8: high and 0.5–0.8: moderate (Brown et al., 2017) and negative values were set to 0 (Fokkema et al., 2017). Since reliability did not differ much between the three methods of MEP estimation, corrected area was used for all further analyses to minimize the effects of bEMG. For each outcome, one-way repeated measures ANOVA was used to determine differences between tasks only for TMS outcomes with at least moderate reliability i.e., ICC >0.5. For the ANOVAs, Greenhouse-Geisser correction was applied when sphericity was violated and Bonferroni’s *post hoc* tests were used for pairwise comparisons. If excitability was not normally distributed in one or more tasks, log transformed values were used for the ANOVA. Spearman’s rank correlation coefficients were used to test for linear associations between neural excitability and ML COP velocity with data pooled across all reliable tasks. Additionally, correlation between neural excitability and bEMG was estimated to test whether any differences in excitability between tasks was confounded by bEMG changes. All descriptive data are presented as mean (±SD).

## Results

### Participant Characteristics

Participants’ age, height and body mass were as follows: reliability group: 22.1 (±2.0) years, 1.75 (±0.10) m and 73.2 (±12.12) kg; task difficulty group: 22.3 (±1.7) years, 1.73 (±0.08) m and 71.5 (±17.24) kg. Only one participant in each group was left-leg dominant.

### Reliability of Responses to TMS in the Four Standing Tasks

ICC values ranged from 0 to 0.96 for peak-to-peak estimates, from 0 to 0.95 for area estimates and from 0 to 0.96 for area estimates normalized to baseline (Table 1). The Bonferroni corrected *p*-value for all the pairwise corrections was less than 0.001. All variables had ICCs >0.5 in wide and narrow stance. In tandem and one leg, reliability varied across the different outcome variables, being highest for LICI (ICC > = 0.75). MT was highly reliable (ICC = 0.98).

**Table 1:** Intraclass correlation coefficients (2,k) for transcranial magnetic stimulation (TMS) outcomes.

*n = 15*	Wide	Narrow	Tandem	One leg
**Peak to peak**				
MEP	0.57*	0.73*	0.53*	0.67*
SICI	0.55*	0.57*	0	0.56*
LICI	0.94**	0.96**	0.85**	0.75*
ICF	0.85**	0.65*	0.30	0.49
**Area**				
MEP	0.61*	0.67*	0.61*	0.74*
SICI	0.66*	0.57*	0	0.50
LICI	0.92**	0.95**	0.91**	0.85**
ICF	0.84**	0.77*	0	0.62*
**Corrected area**				
MEP	0.57*	0.70*	0.48	0.76*
SICI	0.63*	0.64*	0	0.09
LICI	0.96**	0.96**	0.81**	0.78*
ICF	0.73*	0.64*	0	0
MT ICC(2,1)	0.98**			

**High reliability, *Moderate reliability. MT, motor threshold; MEP, motor evoked potential; SICI, short interval intracortical inhibition; LICI, long interval intracortical inhibition; ICF, intracortical facilitation. ICC: >0.8 high; 0.5–0.8 moderate reliability.

### Effects of Task Difficulty on Neural Excitability

**Figure 1:**
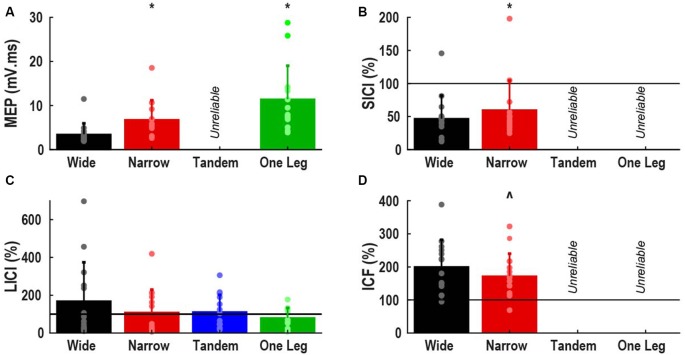
Effect of task difficulty on neural excitability. **(A)** Motor evoked potential (MEP), expressed as area normalized to background EMG; **(B)** short interval intracortical inhibition (SICI); **(C)** long interval intracortical inhibition (LICI); **(D)** intracortical facilitation (ICF). **(B–D)** Expressed as percentage of control MEP, with values greater than 100% indicating facilitation. Bars represent mean and standard deviation, dots represent individual participant data. Horizontal lines indicate size of control MEP. *Different from wide stance (*p* < 0.05). ^∧^Trend for difference with wide stance (*p* = 0.07).

Figure 1 shows the effects of task difficulty on neural excitability (area estimates normalized to baseline) only for variables with ICC >0.5, i.e., at least moderate reliability. MEP size increased by 267% from wide to one leg stance (*p* < 0.001). In narrow compared to wide stance, SICI was lower (*p* = 0.03) and there was a trend for lower ICF (*p* = 0.07).

**Figure 2:**
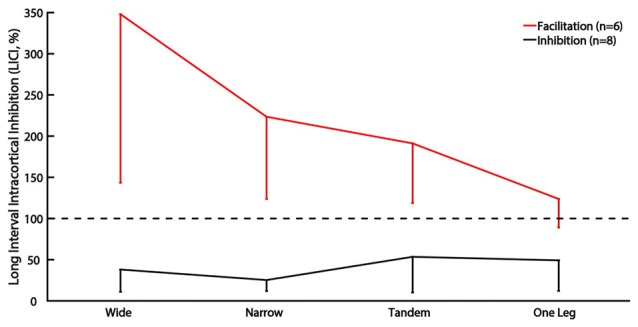
Sub-group analysis of LICI for subjects showing facilitation vs. inhibition. The vertical bars represent standard deviation. Horizontal line indicates size of control MEP, values greater than 100% indicate facilitation.

The main analysis did not reveal any effect of task difficulty on LICI. However, 6 of 14 participants demonstrated atypical facilitation in wide stance (>100%) and often in the other tasks as well. Of the remaining eight participants, only one demonstrated facilitation in the tandem and one leg stance. When participants were divided into facilitation (*n* = 6) and inhibition sub-groups (*n* = 8) and group was used as a between subject factor, we found a significant group by task interaction (*p* = 0.04). Facilitation decreased, and inhibition increased as task difficulty increased in the facilitation sub-group but there were no effects of task difficulty in the inhibition sub-group (Figure 2).

bEMG was 2.3 ± 1.3, 2.3 ± 1.1, 4.3 ± 2.2 and 9.0 ± 2.6% MVC in wide, narrow, tandem and one leg stance, respectively (*p* < 0.001).

### Associations Between Neural Excitability and COP Velocity

**Figure 3:**
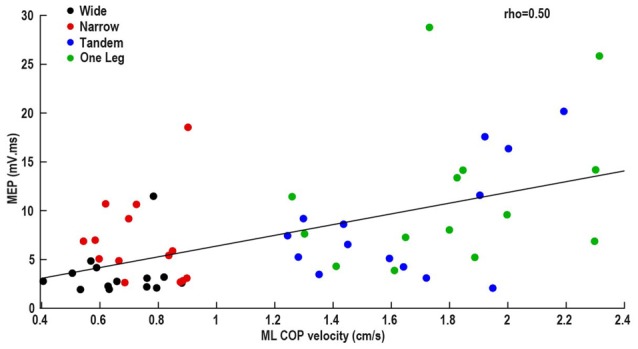
Association between mediolateral (ML) center of pressure velocity and MEP.

In data pooled across wide, narrow and one leg, MEP was correlated with ML COP velocity (ρ = 0.50, *p* = 0.001; Figure 3), and bEMG (ρ = 0.37, *p* = 0.02). Other correlations between neural excitability and COP velocity or bEMG were not significant (all *p* > 0.05). Also, there was a main effect of task on ML COP velocity (*p* < 0.001), with velocity being lowest in wide and highest in one leg.

### Associations Between Excitability in Wide Stance, and Difference Between Wide and More Difficult Tasks

**Figure 4:**
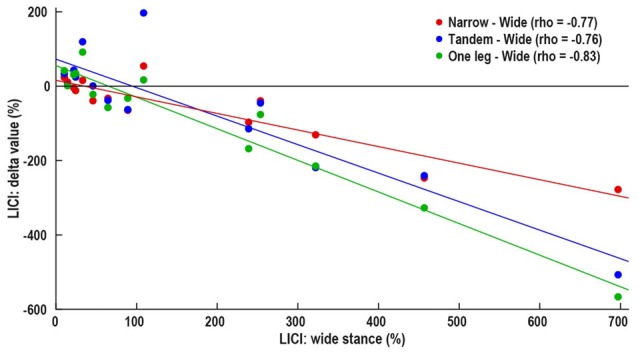
Association between control i.e., wide stance LICI and difference in between LICI wide stance and more difficult conditions—narrow (red), tandem (blue) and one leg (green). Horizontal line represents no change in LICI, above line—decrease in LICI with increasing task difficulty, below line—increase in LICI with increasing task difficulty.

**Figure 5:**
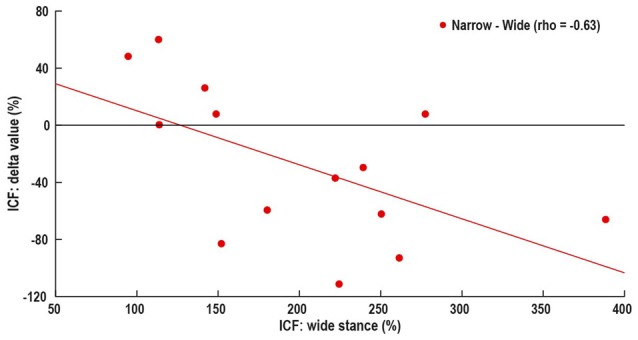
Association between control i.e., wide stance ICF and difference in ICF between wide and narrow stance. Horizontal line represents no change in ICF, above line—increase in ICF with increasing task difficulty, below line—decrease in ICF with increasing task difficulty.

Wide stance LICI was correlated with the differences in LICI between wide and –narrow (ρ = −0.77, *p* = 0.001), tandem (ρ = −0.76, *p* = 0.002), and one leg (ρ = −0.83, *p* < 0.001; Figure 4). ICF measured in wide stance was correlated with difference in ICF between wide and narrow (ρ = −0.63, *p* = 0.01; Figure 5). No such associations were found for MEP and SICI.

## Discussion

In this study we aimed to determine the effects of standing task difficulty on PL corticospinal and M1 excitability, and the association between excitability and COP velocity, in healthy young adults. In partial support of the hypothesis, we found that both corticospinal and M1 excitability responded to the manipulation of task difficulty, but only MEP (i.e., corticospinal excitability) correlated with COP velocity. These data suggest that corticospinal excitability relates to ML sway control as task difficulty increases but cannot confirm whether ML compared to AP sway requires greater active neural control. Additionally, in line with previous findings about TA excitability, both ICF and LICI changes suggested decreasing M1 excitability with increasing difficulty. Therefore, we also discuss an alternative interpretation that M1 excitability reflects other aspects of postural control, including preparation to respond to anticipated perturbations produced by TMS itself. Also, there was large individual variation in the responses to TMS in the four tasks and we discuss how participant-specific intrinsic excitability influences the neural response to increasing difficulty.

### PL Neural Excitability: Effects of Task Difficulty and Association With COP Velocity

During unperturbed standing, neural inputs presumably tune ankle muscle contractions and contribute to sway control. Unlike AP sway (Papegaaij et al., 2014), increasing ML sway correlates with increasing TA (ρ = 0.68; Nandi et al., 2018) and PL (ρ = 0.50) MEP. Increasing MEP could underlie the increase in PL bEMG from ~2% (wide stance) to 9% MVC (one leg stance) and consequently influence sway. However, contrary to our expectations and previous TA findings (Nandi et al., 2018), M1 excitability did not correlate with COP velocity. Therefore, as task difficulty increases corticospinal contribution to ML sway control increases proportionally, but the role of M1 remains uncertain. Since our methods did not exhaustively test all M1 neuron groups (and/or neurotransmitters), we cannot entirely rule out the possibility that M1 excitability contributed to MEP changes. Alternatively, inputs to the spinal alpha motor neuron pool from peripheral afferents, cerebellum or other brain areas can be reflected in the MEP. Further studies should examine MEP in conjunction with other outcomes like H-reflex, to determine what neurophysiological processes and brain areas contribute to the observed increase in corticospinal excitability. In agreement with the TA and soleus data (Papegaaij et al., 2014, 2016; Nandi et al., 2018), SICI in PL decreased with increasing standing difficulty, while our LICI and ICF findings suggested a decrease in neural excitability with increasing difficulty. Previous findings about ICF (Soto et al., 2006; Papegaaij et al., 2014, 2016; Nandi et al., 2018) are equivocal and LICI has not been examined in standing. Additionally, the lack of correlation between M1 excitability and bEMG excludes bEMG as a confounding factor. Therefore, we suspect that M1 excitability changes are not directly related to sway control. We propose an alternative hypothesis that M1 excitability in difficult tasks is involved in other aspects of postural control besides setting muscle activation and controlling sway. Perhaps M1 excitability is tuned by inputs from other brain areas and reflects sensorimotor integration and/or cognitive influences on motor output. Since the TMS pulse creates a mechanical perturbation, we also discuss the possibility that cortical excitability changes are related to preparation and planning to control the anticipated instability.

As the BOS decreases, in addition to increasing sway velocity, the risk of losing balance due to a TMS pulse-induced perturbation increases. Therefore, neuromotor preparation appropriate for each task is required for maintaining balance in anticipation of the TMS pulse-induced perturbation. Perhaps the reduction in SICI we observed is related to this preparatory state. This is possible because cortical inhibition decreases in leg muscles before anticipated postural perturbations (Wälchli et al., 2017) and before muscle contraction in upper extremity muscles. Thus, a release of GABA mediated inhibition is related to movement preparation (Sinclair and Hammond, 2008, 2009; Duque and Ivry, 2009). However, the higher LICI in difficult tasks, observed in a sub-group of participants, contradicts the expected release of inhibition. Though SICI and LICI reflect GABAa and GABAb receptor activity, respectively, the underlying mechanisms interact with each other and SICI is reduced in the presence of LICI (Sanger et al., 2001). Consequently, increased LICI could in fact contribute to an overall decrease in inhibition. However, simultaneous decrease in SICI and LICI has also been reported (Opie et al., 2015) and we cannot rule out the possibility that the increase in LICI serves a distinct purpose. Also, the behavioral implication of lower ICF in difficult tasks is unclear. Though we cannot make direct inferences about the link between neural excitability and behavioral response to the TMS induced perturbation, we present some theoretical possibilities to be tested in future studies. During movement preparation “proactive” inhibition guides the selection of appropriate movement patterns, thereby improving response speed and accuracy (Duque et al., 2017). Additionally, “surround” inhibition is required for appropriate co-ordination between muscles (Sohn and Hallett, 2004). High LICI and low ICF in difficult tasks may contribute to proactive and surround inhibition, consequently ensuring optimal response to the perturbation.

In summary, net corticospinal excitability of ankle muscles contributes to ML sway control. However, cortical inhibition and facilitation are not directly related to sway and perhaps reflect other aspects of postural control like cognitive influences and/or preparation to resist the TMS-induced perturbation. Further studies quantifying the behavioral response to perturbations will determine whether low SICI and ICF, and high LICI are indeed related to effective responses to mechanical perturbations in difficult conditions.

### Association Between Control Task Excitability, and Excitability Response to Increasing Task Difficulty

There is growing interest in the idea of participant-specific “intrinsic neural excitability” (Greenhouse et al., 2017) or “neuronal phenotype” (Fedele et al., 2016). These theories suggest that there may be fundamental differences in excitability between participants, driven by factors such as neurotransmitter concentration, synaptic strength and connectivity with other brain areas. These differences likely affect the neural responses to experimental manipulations. Our data showing that subjects with high LICI and ICF in the easiest condition demonstrate the largest response to task difficulty manipulation, may be an example for unique “neuronal phenotype” (Figures 4, 5). In fact, the increase in LICI is observed only in a sub-group of participants demonstrating atypical facilitation (Figure 2). That is, each participant’s intrinsic excitability influences the response to increasing difficulty, a finding that is masked when differences between tasks are examined only at the group level. Further studies will determine if participant-specific intrinsic excitability is related to behavioral outcomes.

### Atypical Responses to LICI Protocol

A sub-group of participants (*n* = 6) demonstrated facilitation instead of the expected inhibition in LICI. Many healthy participants exhibit atypical facilitation in response to SICI protocols (Marneweck et al., 2011) and our LICI data extend these findings. LICI primarily suppresses the late I-waves generated by magnetic stimulation (Di Lazzaro et al., 2002) and there can be substantial variation in the “efficiency of late I-wave recruitment” between participants (Hamada et al., 2012). It is possible that the LICI protocol does not elicit inhibition in participants in whom TMS does not evoke late I-waves. However, the underlying reasons for inter-individual differences in I-wave recruitment efficiency are unknown. Additionally, we cannot rule out the possibility that the stimulation intensities and ISI used in this study engages different neuron populations in different groups of participants. Indeed, it is possible that in the facilitation sub-group the intended GABAb inhibitory neurons are not activated. Though there is some evidence that TMS responses are influenced by genetic variations (Mori et al., 2011) or strength of connectivity between brain areas (Fedele et al., 2016), future studies will determine what individual characteristics explain LICI variability.

### Reliability of PL Neural Excitability

The main analyses were guided by reliability measurements in each of the four standing postures. We found ICCs ranging from 0.53 to 0.76 for MEP, 0–0.66 for SICI, 0.75–0.98 for LICI and 0–0.85 for ICF. For all subsequent analyses, we compared only the tasks and variables with at least moderate reliability i.e., ICC >0.5 (Brown et al., 2017). This approach has not previously been employed in postural control studies but is necessary, considering that unreliability of TMS outcomes can confound conclusions (Beaulieu et al., 2017). In sitting, PL MEP reliability ranges from 0 to 0.98 (Luc et al., 2014). In standing, sway-related fluctuations are superimposed on sustained muscle contractions, potentially increasing inter-trial MEP variability and decreasing reliability, especially in difficult tasks. Indeed, we found the greatest sway and lowest reliability in tandem stance. Independent of task difficulty, LICI was the most reliable TMS outcome, possibly due to a lower susceptibility to bEMG fluctuations (Wassermann et al., 1996) compared to other TMS outcomes. Finally, we compared different methods of MEP estimation since leg muscle MEPs are often polyphasic and may be better characterized by area measures, which account for both amplitude and duration. However, we found similar ICCs for peak-to-peak, area, and area-adjusted for bEMG. Therefore, we used the latter for the main analysis, to minimize the influence of inter-trial bEMG fluctuations.

### Limitations and Conclusions

Majority of the participants in this study were female, limiting the generalizability of the results. Further investigations are required to determine if there are any sex differences. Another limitation is that manual positioning of the coil may have contributed to the inter-trial MEP variability. However, since TMS has limited spatial resolution, we do not expect the main results and conclusions to change if neuro-navigation is used.

In summary, there is a correlated increase in leg muscle corticospinal excitability and COP velocity as ML BOS decreases. Cortical inhibition and facilitation decrease with decreasing ML BOS but do not correlate with sway, suggesting that cortical excitability reflects other postural goals besides sway control. Correlations between participant-specific intrinsic excitability and neural response to difficulty manipulation, along with the decrease in GABAb inhibition only in participants with atypical facilitation, suggest an interaction between experimental manipulations and individual characteristics.

## Author Contributions

TN, CL, HK, BF, GS and TH: conceptual development. TN, CL, HK, LB, IK and TH: data collection and analysis. TN, CL, HK, BF, GS and TH: manuscript writing.

## Conflict of Interest Statement

The authors declare that the research was conducted in the absence of any commercial or financial relationships that could be construed as a potential conflict of interest.
